# Laboratory findings predictive of critical illness in hospitalized COVID-19 patients in Tunisia

**DOI:** 10.12688/f1000research.151333.2

**Published:** 2024-11-18

**Authors:** Donia Belkhir, Hana Blibech, Line Kaabi, Saoussen Miladi, Mohamed Aymen Jebali, Jalloul Daghfous, Nadia Mehiri, Ahmed Laatar, Nozha Ben Salah, Houda Snene, Bechir Louzir

**Affiliations:** 1 Pulmonology, University Hospital Center Mongi Slim, La Marsa, Tunis, Tunisia; 2 Rheumatology, University Hospital Center Mongi Slim, La Marsa, Tunis, Tunisia

**Keywords:** COVID-19, Viral pneumonia, Critical illness, Adult respiratory distress syndrome, Biomarkers, Prognosis

## Abstract

**Background:**

COVID-19 disease has spread rapidly worldwide, causing high mortality. Accessible biomarkers capable of early identification of patients at risk of severe form are needed in clinical practice. The aim of the study was to determine the biological markers that predict a critical condition.

**Methods:**

Retrospective study including patients with confirmed COVID-19 hospitalized between September 2020 and June 2021. The primary endpoint was progression to critical status within 7 days from admission. We defined two groups:

Critical group: Patients who developed a critical condition or died or transferred to the ICU before or at 7 ^th^ day.

Non-critical group: Patients who remained in non-critical respiratory status until 7 ^th^ day or discharged before or at 7 ^th^ day.

**Results:**

Our study included 456 patients, with a sex ratio of 1.32 and an average age of 62 years. At the 7 ^th^ day of hospitalization, 115 (25.2%) patients were in the critical group and 341 (74.8%) patients were in the non-critical group. The univariate logistic regression indicated that laboratory findings between non-critical and critical groups showed that C-reactive protein (CRP) (p=0.047), D-Dimer (p=0.011), creatinine (0.026), creatine kinase (p=0.039), lactate dehydrogenase (p=0.04), and troponin (p=0.001) were all higher among patients in critical group. However, lymphocyte (p<0.001) and platelet (p<0.001) counts were significantly lower among the critical group. Multivariate logistic regression model, identified four independent risk factors: lymphopenia (OR=2.771, 95%CI=1.482-5.181, p=0.001), Neutrophil to Lymphocyte Ratio (NLR) (OR=2.286, 95%CI=1.461-3.578, p<0.001), thrombocytopenia (OR=1.944, 95%CI=1.092-3.459, p=0.024), and CRP>71.5 (OR=1.598, 95% CI=1.042-2.45, p=0.032) were associated to critical group.

**Conclusions:**

Our results show the predictive value of lymphopenia, thrombocytopenia, high NLR and CRP levels to evaluate the prognosis of COVID-19 pneumonia. A prognostic score could be proposed for guiding clinical care and improving patient outcomes.

## Introduction

In December 2019, a novel coronavirus caused viral pneumonia cases in Wuhan, China, spreading globally. Termed SARS-CoV-2, it was declared a “public health emergency of international concern” in January 2020, impacting various levels. Tunisia reported imported COVID-19 cases in March 2020, with a milder initial wave due to strict measures. The second wave strained care facilities in July 2020 due to eased preventive measures. ^1^ Tunisia’s limited healthcare resources posed challenges, necessitating hospital reorganization and resource optimization. Intensive care units (ICU) expanded, and departments like pulmonology were dedicated to COVID-19 care. Identifying high-risk patients and predictors of severe outcomes has become of paramount importance. Most patients infected with SARS-CoV-2 experience mild to moderate respiratory symptoms, while others may develop severe respiratory distress, septic shock, multi-organ failure, and even death. Studies have shown that vascular processes underlie organ damage in critically ill patients, driven by inflammatory cascades, complement activation, and pro-inflammatory cytokines. These vascular injuries cause edema and acute respiratory distress syndrome (ARDS) in the lungs ^2^^,^^3^ and play a crucial role in cardiovascular (ischemia, thromboembolic complications) and cerebral damage. This highlights the relationship between specific markers of inflammation and vascular injury and the severity of SARS-CoV-2 pneumonia. Urgent identification of predictors guided risk assessment and intervention studies. This contributed to the effective allocation of resources and informed clinical decision-making. Moreover, distinguishing severe cases using parameters like haematological and inflammatory markers improved clinical management. ^4^^,^^5^ This is a study performed at University Hospital Center Mongi Slim, focused on COVID-19 patients hospitalized between October 2020 and June 2021 in the COVID-19 unit comprising Pulmonology and Rheumatology departments. The objectives of this study was to describe the clinical and evolutionary characteristics of hospitalized patients with COVID-19 and to determine predictive biological markers of a critical condition on the 7th day of admission.

## Methods

This was a retrospective study, conducted at University Hospital Center Mongi Slim, including patients hospitalized for COVID-19 pneumonia between October 2020 and June 2021 in the COVID-19 unit: Pulmonology Department and Rheumatology Department, which were jointly managed in collaboration between the two teams.

### Study design

*Inclusion and exclusion criteria* :

Eligible patients were those admitted to the COVID-19 unit between October 2020 and June 2021 with moderate to severe COVID-19 pneumonia at admission, confirmed for SARS-CoV-2 via Reverse Transcription-Polymerase Chain Reaction (RT-PCR), Rapid Antigen Test (RAT), or positive SARS-CoV-2 serology (IgM or IgG) for vaccine-naive patients. All patients were at their first infection.

Patients were not included if they had a mild COVID-19 infection throughout their hospitalization and another reason for hospitalization, if they were patients admitted to the COVID-19 unit after an initial stay in the ICU, or if they remained in the emergency department for more than 7 days before being admitted to the COVID-19 unit.

We excluded patients who were admitted to the COVID-19 unit with hypoxemic pneumonia and suggestive findings of COVID-19 pneumonia on thoracic CT scan but with negative virological results (RT-PCR, RAT, and SARS-CoV-2 serologies), and an alternative cause was identified during the course of the disease. Additionally, patients were excluded if virological documentation data could not be located.

*Outcome measure* :

We defined the development of a critical state before or on the 7th day of hospitalization (D7) as the outcome measure. Patients were categorized into two groups:

1) Critical Group: Event group including:
  • Patients who developed a critical state by D7. The definition of a critical state will be detailed in the following paragraph.
  • Patients who died before or on D7.
  • Patients transferred to the ICU before or on D7.
2) Non-Critical Group: Non-event group including:
  • Patients who maintained a stable and non-critical respiratory state until D7.
  • Patients discharged from the hospital before or on D7.

*Definition of a Critical State* : We defined a critical state as:

• The occurrence ARDS requiring invasive or non-invasive respiratory support. For this criterion, we defined the oxygen requirement level of 10 liters with a high-concentration mask as the threshold for evolving into ARDS. This threshold was considered throughout the COVID unit’s activity based on the medical team’s experience as an indicator of a critical state and warranting alerting the ICU.
• Vital distress or shock, sepsis, and/or organ failure. In all cases, ICU admission is indicated. We relied on the definitions from the World Health Organization (WHO) version of November 2021 ^6^ and the National Evaluation and Accreditation Agency for Health (INEAS) version of April 2021 ^7^ to establish the definition of a critical state.

### Data collection

Data were retrospectively and anonymously collected between June 2023 and January 2024. All demographic, clinical, biological, radiological, therapeutic, and progression-related data were extracted from the medical records. The medical records followed a standardized format with predefined items consistently used in both departments of COVID-19 unit. The collected information included demographic data, clinical details recorded at admission and D7, and biological data obtained at admission. For the latter, all patients underwent a routine standard biological assessment at admission complete blood count (CBC) including leukocytes, lymphocytes, neutrophils, Neutrophil-to-Lymphocyte Ratio (NLR), platelets, hemostasis profile (Prothrombin Time, Activated Partial Thromboplastin Time, D-Dimers) as a hypercoagulability biomarker), renal profile (Urea, Creatinine, Blood Ionogram), liver profile (Aspartate Aminotransferase (AST), Alanine Aminotransferase (ALT)), markers of muscle injury (Creatine Kinase (CK), Lactate Dehydrogenase (LDH)), and C-Reactive Protein (CRP) as a marker of high inflammatory response. Some parameters were unavailable due to reagent shortages, and others were optional based on clinical presentation, such as Troponin (myocardial injury biomarker) and N-terminal prohormone of brain natriuretic peptide (NT-ProBNP); an indicator of cardiac stress or heart failure. Chest computed tomography (CT) data were recorded at admission and upon aggravation during evolution. Therapeutic data included oxygen therapy, antibiotic treatment, corticosteroids, anticoagulation, and other measures, adhering to a uniform protocol guided by INEAS recommendations. Evolvement data covered hospital discharge, transfers to ICU, COVID-19 unit deaths, and complication occurrences.

### Statistical analysis

For the analysis of the association between two qualitative variables, we used Pearson’s Chi-squared test to compare two frequencies when the conditions for its application were met, and the Fisher’s test otherwise. For the analysis of the association between a qualitative variable and a quantitative variable, we employed the Student’s t-test to compare two means in the case of a normal distribution, and the non-parametric Mann Whitney test otherwise. Correlations between two quantitative variables were calculated using the Pearson correlation coefficient, with significance tested bilaterally. In the multivariate study, a binary logistic regression model was followed, and the risk was computed as the Odds Ratio (OR) with a retained 95% confidence interval (CI 95%). To assess performance, we utilized parameters related to the Receiver Operating Characteristic (ROC) Curve, as well as sensitivity and specificity. Pre- and post-test probabilities were determined through likelihood ratios represented on the Fagan nomogram. We set the significance threshold at p ≤ 5%.

### Ethical considerations

This retrospective study received approval from the Ethics Committee of University Hospital Center Mongi Slim, under approval number 57/2023 on Friday, 22 December 2023.

We followed strict ethical committee guidelines that allowed for exemption of consent, due to the non-intrusive nature of the study and the use of non-identified data to ensure confidentiality and anonymity of participants. All personally identifiable data were anonymized prior to analysis to protect individuals’ privacy. We also carefully assessed the risks and benefits of our research, ensuring to minimize the former and maximize the latter for participants and the scientific community. No financial or other conflicts of interest were identified by the authors of this study. Policies regarding the future use of data and their potential sharing with other researchers were strictly established in accordance with ethical guidelines.

## Results

**Figure 1.**
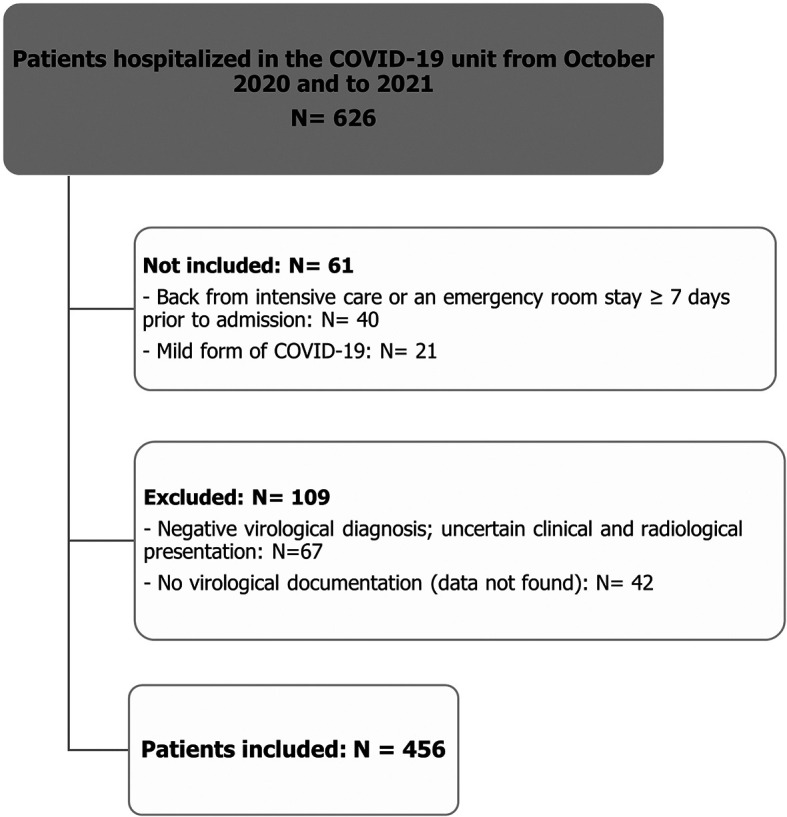
Flow chart illustrating the patient selection process.

Among 626 patients hospitalized in the COVID-19 unit of University Hospital Center Mongi Slim for a period of 9 months (October 2020 - June 2021), we selected 456 patients after applying the inclusion and exclusion criteria. The number of included, not included, and excluded patients is summarized in the flowchart below ( Figure 1).

1) **Patient characteristics**
On the 7th day of hospitalization, 115 (25.2%) evolved to a critical condition. Among the patients in the study, 260 (57%) were males; 196 (43%) were females. The gender of the patients was not statistically significant between critical and non-critical groups (p=0.106). As shown in Table 1, Chi-square outcome indicated that the age differed significantly between the critical and non-critical patients (p=0.004). In fact, the mean age for the non-critically ill patients was 61.5 ± 13.2 years while the critically ill patients had a mean age of 65.7 ± 14. Chronic concomitant diseases were found among 322 patients (70.6%), with no significant difference between both groups. The mean time from onset of symptoms to admission into the COVID-19 unit was 8.5 ± 4.12 days. This lapse was significantly shorter for the critically ill patients: 7.9 vs 8.8 days (p=0.022). Among the total cases, 31.4% had an extent of lesions greater than 50% on chest CT, the critical group had a higher proportion of cases with an extent exceeding 50% (37% versus 25.6%, p=0.021).
2) **Laboratory test outcomes**
The CBC findings shown in Table 2 indicate that concentration of the lymphocytes were statistically lower among critically ill patients (0.85 ± 0.24) compared to non-critically ill patients (1.10 ± 0.33, p<0.001). Lymphopenia also occurred among 89.5% of patients in critical group (p=0.002) ( Table 3). Similarly, the platelet count was lower among the critical group (203 vs 235, p<0.001). The level of WBC and neutrophil did not statistically differ between both groups.
  The mean NLR was 7.52. The NLR at admission was higher in the critical group with a marginally significant difference (p=0.073). There was also a significant correlation between the NLR and the extent of lesions on the chest CT (r=0.213, p<10 ^−3^).
  The CRP concentration outcome is the other biochemistry test that was statistically significantly different between critically ill (122 ± 78) and non-critically ill patients (106 ± 72, p=0.047). Upon admission, data regarding levels of D-dimer (full data available for 136 patients), hypersensitive troponin (full data available for 117 patients), B-type natriuretic peptide (full data available for 67 patients) were obtained. As shown in Table 2, in comparison to the non-critically ill patients, patients with critical illness had significantly higher levels of D-Dimer, hypersensitive and NT-pro-BNP. Besides, patients with severe pneumonia had higher rates of rhabdomyolysis markers than patients exhibiting less critical forms of the disease; (CK: 127 vs 83, p=0.039), (LDH: 831 vs 701, p=0.04). However, the findings presented in Table 2 indicate that the concentration of AST and ALT did not vary between both groups.

**Table 1.** Baseline characteristics of 456 patients infected with COVID-19 pneumonia on admission.

Characteristics	Total number (N=456)	Non-critical group (N=341)	Critical group (N=115)	p-value
**Gender**				0.106
Male N, (%)	260 (57)	187 (55)	73 (64)	
Female N, (%)	196 (43)	154 (45)	42 (36)	
**Age, mean, SD (years)**	62.56 ± 13.5	61,5 ± 13.2	65,7 ± 14	**0.004**
**Onset of symptoms to admission, mean, SD ( days)**	8.5 ± 4.12	8.8 ± 4.12	7.9 ± 4.11	**0.022**
**BMI, kg/m** ^**2**^ **, SD**	29.3 ± 5	29.33 ± 5.45	29.06 ± 5.88	0.672
**Chronic concomitant diseases, N (%)**	322 (70.8)	236 (69.2)	86 (75.4)	0.256
Hypertension, N (%)	198 (43.4)	146 (42.8)	52 (45.6)	0.653
Diabetes, N (%)	171 (37.5)	126 (37)	45 (39.5)	0.676
Dyslipidemia, N (%)	72 (15.8)	51 (15)	21 (18.4)	0.401
Coronary artery disease, N (%)	45 (9.9)	35 (10.3)	10 (8.8)	0.626
Obesity, N (%)	203 (40)	157 (46)	46 (40)	0.298
COPD, N (%)	23 (5)	16 (4.7)	7 (6.1)	0.554
**Symptoms**				
Asthenia, N (%)	324 (71)	238 (70.8)	86 (74.8)	0.308
Fever, N (%)	286 (63)	211 (62.6)	75 (65.2)	0.522
Myalgia, N (%)	221 (48.5)	179 (53.3)	42 (36.5)	**0.003**
Dyspnea, N (%)	305 (67)	226 (67.3)	79 (68.7)	0.633
Cough, N (%)	229 (50)	169 (50.3)	60 (52.2)	0.628
Nasal symptoms , N (%)	25 (5.5)	14 (4.2)	11 (9.6)	**0.026**
**Peripheral oxygen saturation in room air, %, SD**	88 ± 4.24	88.8 ± 4	87.1 ± 4.5	**<0.001**
**CT findings**				
Extent of lesions > 50%, N (%)	129 (31.4)	86 (25,6)	43 (37)	**0.021**
Bilateral distribution, N (%)	386 (98.2)	287 (98.6)	99 (97)	0.42
Ground-glass opacity, N (%)	385 (96.7)	283 (93)	102 (95)	0.568
Consolidations, N (%)	276 (70.6)	202 (69.7)	74 (73.3)	0.662
Pleural effusion, N (%)	27 (6.9)	17 (6)	10 (9)	0.183
Pericardial effusion, N (%)	40 (10.2)	23 (8)	17 (16)	**0.013**
**Outcome**				
Transfer to intensive care unit, N (%)	81 (16.6)	11 (3.2)	70 (60.9)	**<0.001**
Deaths in the COVID-19 unit, N (%)	22 (4.8)	1 (0.3)	21 (1.3)	**<0.001**

**Table 2.** Laboratory findings among critical and non-critical COVID-19 patients on admission to hospital.

Variables	Normal range	Total number (N=456)	Non-critical group (N=341)	Critical group (N=115)	p-value
Leukocyte, × 10 ^9^/L	4-10	8.1 ± 3.8	7.4 ± 2.4	7 ± 2	0.4
Lymphocyte, × 10 ^9^/L	1.5-4	1.1 ± 0.6	1.1 ± 0.33	0.8 ± 0.24	**<0.001**
Neutrophil, × 10 ^9^/L	1.5-7	6.5 ± 3.7	5.7 ± 0.19	5.8 ± 0.38	0.926
Platelet, × 10 ^9^/L	150-400	252 ± 114	235 ± 6.53	203 ± 8.76	**<0.001**
NLR (ratio)	-	7.52 ± 8.05	7.12 ± 0.43	8.69 ± 0.76	0.073
C-reactive protein, mg/L	<8	110 ± 73	106 ± 71.7	122 ± 78.2	**0.047**
D-dimer (N=136), μg/L	0-500 if < 50 years 0-(age*10) if > 50 years	1214 ± 1311	712 ± 1401 (N=99)	1027 ± 1035 (N=37)	**0.011**
Creatinine, μmol/L	50-101	86.6 ± 62.5	85 ± 64.3	91.6 ± 56	**0.026**
Blood urea nitrogen, mmol/L	2.5-7	7.8 ± 6	6.2 ± 6.3	7 ± 4.4	0.061
Creatine Kinase, U/L	<168	146.7 ± 168	83 ± 153	127 ± 199	**0.039**
Lactate Dehydrogenase, U/L	125-222	784.7 ± 34	701 ± 342	831 ± 334	**0.04**
Aspartate aminotransferase, U/L	5-40	40.5 ± 31	39 ± 26.25	45 ± 40.6	0.172
Alanine aminotransferase, U/L	5-45	34.7 ± 29	34 ± 26.8	36 ± 34.5	0.740
Natremia, mmol/L	135-145	134.7 ± 4.5	135 ± 4.1	133 ± 5.5	**0.011**
Kalemia, mmol/L	3.5-5	4.09 ± 0.6	4.12 ± 0.56	3.98 ± 0.63	**0.029**
Hypersensitive troponin (N=117), ng/L	<19	180.2 ± 1570	15.5 ± 57.2 (N=86)	637 ± 3036 (N=31)	**0.001**
NT-Pro-BNP (N=67), pg/mL	<125	989.7 ± 1149	779 ± 1315 (N=41)	1322 ± 1608 (N=26)	**0.011**

**Table 3.** Comparison of biological abnormalities between critically and non-critically ill COVID-19 pneumonia patient groups.

Biological abnormalities	Total number (N=456)	Non-critical group (N=341)	Critical group (N=115)	p-value
Hyperleucocytosis, N (%)	110 (24.1)	80 (23.5)	30 (26.1)	0.569
Lymphopenia, N (%)	355 (77.9)	253 (76)	102 (89.5)	**0.002**
Neutrophilia, N (%)	155 (34.6)	113 (34)	42 (36.8)	0.573
Thrombocytopenia, N (%)	59 (12.9)	37 (11.1)	22 (19.5)	**0.023**
Increased CRP, N (%)	424 (93.5)	315 (96)	109 (98.2)	0.375
D-Dimer > 1000 ng/ml, N (%)	50 (40.8)	31 (31.3)	19 (51.4)	**0.031**
Increased blood urea nitrogen, N (%)	186 (40.8)	135 (39.6)	51 (44.3)	0.369
Increased aminotransferase, N (%)	157 (39.5)	116 (39.7)	41 (39)	1
Increased Creatine Kinase, N (%)	55 (24.2)	31 (18.6)	24 (40)	**0.001**
Increased Lactate dehydrogenase, N (%)	190 (41.6)	135 (39.5)	55 (47.8)	1
Increased tropnin, N (%)	21 (18)	9 (10.5)	12 (38.7)	**<0.001**

### Univariate analysis

The univariate logistic regression indicated that laboratory findings between non-critical and critical groups showed numerous differences including C-reactive protein (p=0.047), D-Dimer (p=0.011) and creatinine (0.026) as well as, creatine kinase (p=0.039), lactate dehydrogenase (p=0.04), troponin (p=0.001) and NT-pro-BNP level (p=0.011) which were all higher among patients in critical condition. On the other hand, lymphocyte (p<0.001) and platelet (p<0.001) counts, natremia (p=0.011) and kalemia (p=0.029) were significantly lower among the critical group, whereas liver enzymes showed no significant differences. Table 3 summarizes the biological abnormalities on admission.

### Multivariate analysis

Based on the multivariate logistic regression model, four variables were demonstrated as independent risk factors. As shown in Table 4, the results indicated that lymphopenia (OR=2.771, 95%CI=1.482-5.181, p=0.001), NLR (OR=2.286, 95%CI=1.461-3.578, p<0.001), thrombocytopenia (OR=1.944, 95%CI=1.092-3.459, p=0.024), and CRP>71.5 (OR=1.598, 95% CI=1.042-2.45, p=0.032) were associated with critical outcome.

**Table 4.** Multivariate analysis of biological markers predicting critical condition.

Parameters	OR	95%CI	p-value
Lymphopenia	2.771	1.482-5.181	**0.001**
NLR > 5	2.286	1.461-3.578	**<0.001**
Thrombocytopenia	1.944	1.092-3.459	**0.024**
CRP > 71.5 mg/L	1.598	1.042-2.45	**0.032**

**Figure 2.**
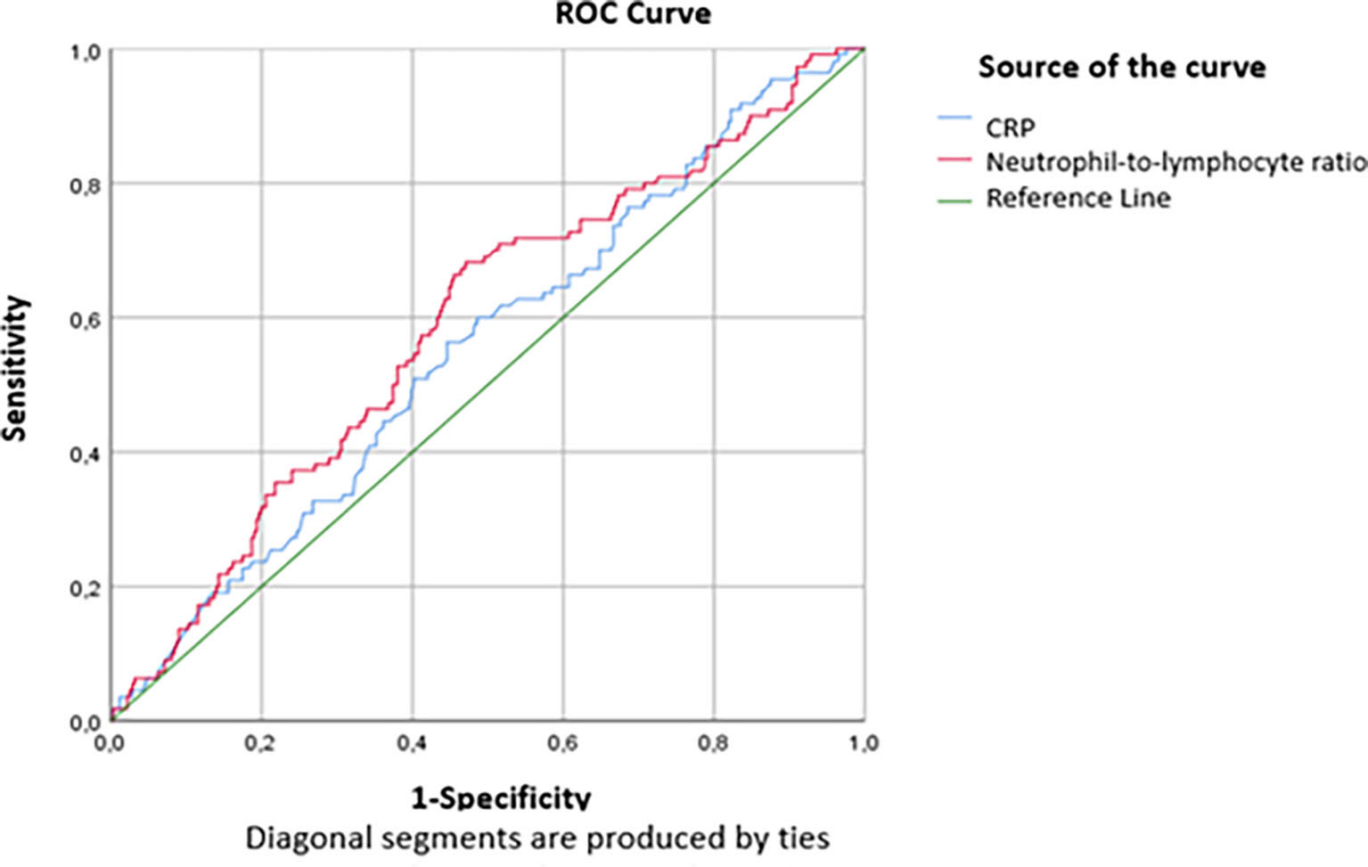
ROC curve indicating the performance of CRP and NLR in predicting critical cases.

**Figure 3.**
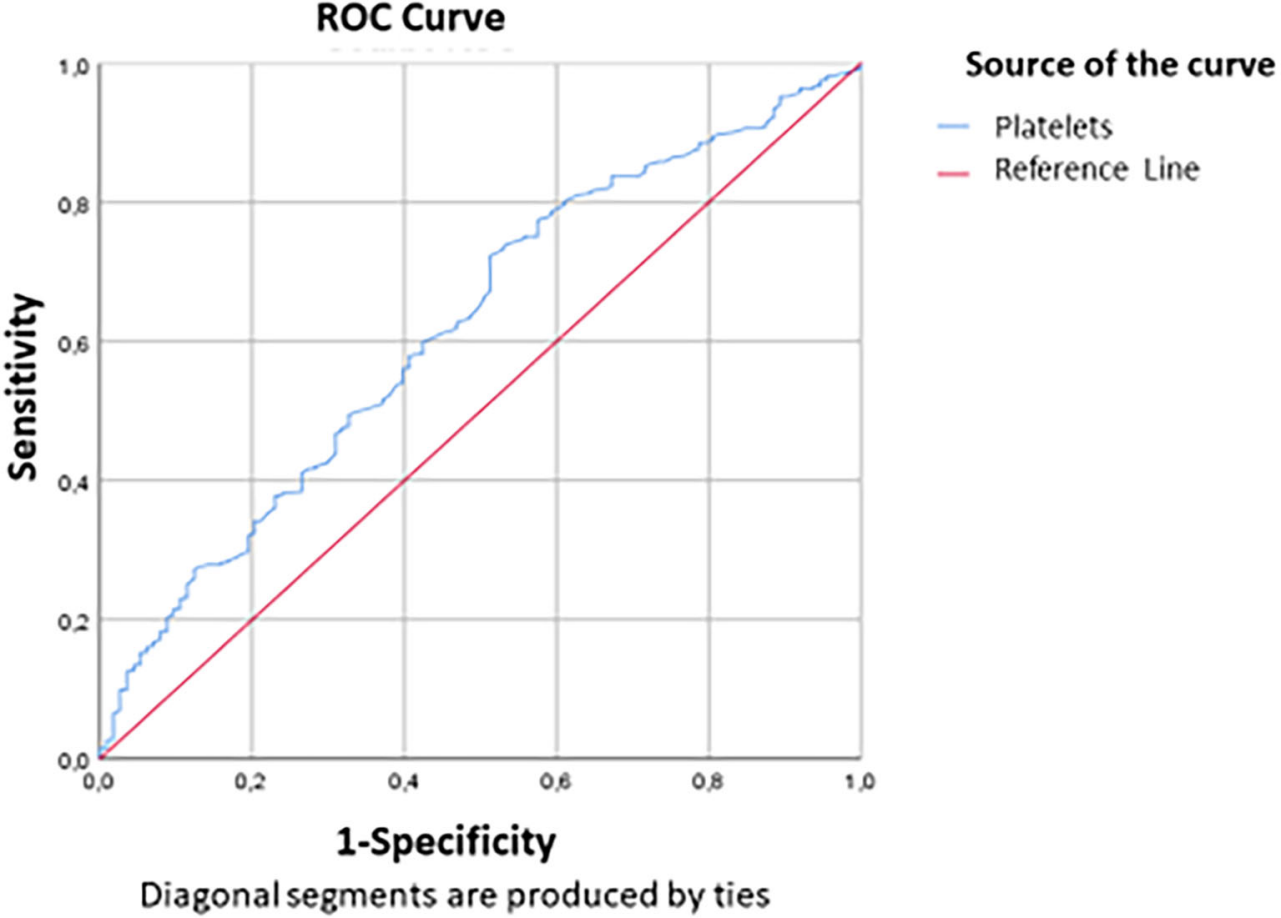
ROC curve indicating platelet count performance in predicting non-critical cases.

The threshold values of 71.5mg/L for the CRP level and 5 for the RNL were defined according to the study of the ROC curve according to the critical and non-critical groups. The findings in Table 4 show that statistically significant AUCs were obtained for CRP (AUC=0.533, p=0.032) and NLR (AUC=0.589, P<0.001). Thus, the performance of the NLR was slightly superior to that of the CRP in predicting a critical condition on day 7 ( Figure 2). For a threshold equal to 71.5 mg/L, the sensitivity and specificity of CRP to predict critical illness were respectively equal to 66.1% and 38.1%, while for a threshold equal to 5, the sensitivity and specificity of the NLR to predict the critical illness were respectively equal to 68.7% and 51% ( Table 5). Figure 2 and Figure 3 show the area under the ROC curve of CRP and NLR ( Figure 2) and platelet count ( Figure 3). The platelet count was negatively correlated with the occurrence of a critical condition and it was quite efficient in predicting non-critical cases; (AUC=0.62; CI95%=0.56-0.679) ( Figure 2). The best threshold was estimated at 187.5*10 ^3^ elements/mm ^3^ corresponds to a sensitivity and specificity equal to 72.4% and 48.7% respectively with a quite good accuracy ( Table 6).

**Table 5.** Performance parameters of CRP and NLR.

	Cut-off	AUC (95%CI)	Sensitivity	Specificity
CRP (mg/L)	71.5	0.533 (0.492-0.614)	66.1%	38.1%
NLR	5	0.589 (0.528-651)	68.7%	51%

AUC: Area under ROC curve.

**Table 6.** Performance parameters of platelet count.

	Cut-off	AUC (95%CI)	Sensitivity	Specificity
Platelets (10 ^9^/L)	187.5	0.62 (0.56-0.679)	72.4%	48.7%

AUC: Area under ROC curve.

## Discussion

Based on the univariate analysis of our study, some biomarkers were predictive of the progression to a critical state on the 7th day of hospitalization for COVID-19 pneumonia.

Most predictive models are based on static demographic characteristics and clinical parameters obtained at admission. Understanding biomarkers associated with COVID-19 severity and their kinetics can contribute to the development of more accurate predictive models of disease progression. According to Lasso and al.'s ^8^ study, a chronological analysis of biomarkers namely, anti-SARS-CoV-2 IgG, leukocyte, neutrophil, and lymphocyte counts, CRP, and urea levels, showed a correlation with disease severity and mortality. IgG increased between weeks 1 and 3 in all cases. Leukocyte and neutrophil counts were higher in non-survivors from days 7 to 32; urea stayed elevated from days 6 to 43. CRP levels increased significantly above 10 mg/dl during the first 4 days in non-survivors and remained significantly higher between days 5 and 28. Studies have shown the variation over time of the various markers, particularly the inflammatory ones accompanying the “cytokine storm” that precedes the onset of ARDS, which usually occurs after 8 to 12 days from the onset of symptoms. ^2^^,^^9^ Hence, we chose the 7th day after hospitalization, approximately corresponding to the 14th day after symptom onset, as the threshold for classifying patients into critical or non-critical groups.

Analysis of demographic characteristics revealed that critically ill patients were older than non-critically-ill ones which aligns with previous studies’ results. ^10^ However, this age difference was smaller compared to that reported previously, noted as 20 years. ^11^ Advanced age was also an important predictor of mortality in Middle East Respiratory Syndrome (MERS). ^12^

We underline the fact that the study sample of COVID-19 patients cared during the study involved more males than females, as well among both groups (critical and non-critical), which supports previous observations that indicate gender as a potential determinant of likelihood to develop serious complications and unfavorable outcome. In fact, a multicenter study conducted in China, including 1099 patients hospitalized for COVID-19 pneumonia in 552 hospitals, ^13^ had shown a clear male predominance (58%). This male susceptibility has been clearly elucidated in an Italian study, ^14^ in which, two significant factors incriminated in the initiation of viral infection were emphasized, namely angiotensin-converting enzyme 2 (ACE2) and transmembrane serine protease 2 (TMPRSS2), both of which are influenced by gender.

Moreover, this observed difference could also be attributed to behavioral differences between the sexes, notably that smoking and alcohol consumption are more often seen among men ^15^ heightening the risk of chronic comorbidities such as cardiovascular and chronic pulmonary diseases, which have been associated with a greater likelihood of developing severe illness with COVID-19. ^16^

### Hematological biomarkers

Our univariate analysis findings regarding the CBC parameters were different to previous findings. ^17^^,^^18^ Previous studies concluded that increased neutrophils count is characteristic of patients with severe cases. ^19^ Evidence suggests that severe COVID-19 is associated with elevated neutrophil levels, which increase inflammation via cytokine storm, and hemorrhages especially in the lungs, which occur as a result of neutrophil-induced tissue damage.

According to our data, lymphopenia occurred in 78% of cases. The lymphocyte count ranged between 500 and 1000/mm ^3^ among 36.6% of our patients while 8.8% had deep lymphopenia (< 500/mm ^3^). Our results also showed, using the multivariate logistic regression model, that lymphopenia was an independent risk factor for a severe illness. These findings are in line with the literature. Indeed, several studies have assessed the impact of lymphopenia on disease severity. For example, Zhang and al. ^20^ reported an average blood lymphocyte count of 1200/mm ^3^, and Jiang and al. ^11^ showed that 41% of patients had a lymphocyte count of less than 1000/mm ^3^. This count was significantly lower in patients with an unfavorable outcome. Additionally, Bellan and al. ^21^ demonstrated that a low lymphocyte count at admission was correlated with the risk of mortality due to SARS-CoV-2 infection. Two Chinese meta-analyses ^22^^,^^23^ have concluded that patients with severe COVID-19 pneumonia had lower blood lymphocyte counts compared to non-severe forms, and lymphopenia was associated with a three-fold higher risk of severe infection, particularly ARDS. When viral particles resulting from the replication of SARS-CoV-2 spread through the respiratory mucosa, it leads to a cytokine storm, generating a cascade of immune responses primarily involving CD4 and CD8 T lymphocytes. ^24^

In our study, thrombocytopenia was present among 13% of cases and was correlated with the progression to a critical condition (p=0.023). We demonstrated that thrombocytopenia was associated with an odds ratio (OR) of 1.94 of developing a critical condition. Our findings are consistent with those of the literature. Indeed, a meta-analysis of 21 studies including 3,377 SARS-CoV-2 positive patients ^4^ found that platelet count was negatively correlated with the severity of the infection as well as mortality. Another meta-analysis involving 1,779 COVID-19 patients ^25^ revealed that thrombocytopenia was associated with a threefold higher risk of severe disease, and mortality. Moreover, the platelet count was also correlated with disease severity scores and the risk of mortality in ICU. ^26^^,^^27^ Platelet count was considered as an independent risk factor for COVID-19 mortality. This count was significantly lower in COVID-19 patients who died compared to survivors. ^21^^,^^28^ Platelet kinetics could also help to identify patients at risk of an unfavorable outcome. ^29^ Similarly, Choi KW et al. ^30^ reported in a study evaluating the prognosis of SARS infection in 2003, that lymphopenia and thombopenia were found in 73% and 50% of cases respectively.

In COVID-19 patients, thrombocytopenia appears to be multifactorial. In the case of ARDS, there is a significant platelet consumption resulting from the combination of viral infection and mechanical ventilation, leading to endothelial damage and platelet activation, aggregation, and thrombus formation in the lungs. ^25^^,^^29^ Moreover, the lung can be a site for platelet release from mature megakaryocytes, and alteration of the pulmonary capillary bed can affect platelet degranulation and pulmonary megakaryocytopoiesis. ^25^^,^^29^

In our study, the mean NLR was 7.52. This ratio was higher in the critical group patients (p=0.073). As we previously mentioned, lymphopenia was more common among patients in a critical condition. It appears logical to observe a trend of increasing NLR in severe forms. This finding was consistent with studies of both Yang and al. ^31^ and Yan and al., ^32^ where a higher NLR was correlated with severe forms and mortality, respectively (p<10 ^−3^). NLR is a biomarker that integrates two subtypes of leukocytes representing two inversely related immune pathways. It provides information about systemic inflammation and is a useful biomarker for predicting bacterial infection, including pneumonia, better than absolute leukocyte, lymphocyte, or neutrophil counts. ^32^ A high NLR reflects a discrepancy in the inflammatory response. ^32^ Numerous studies and meta-analyses ^33^^–^^35^ have proved that an increased NLR is associated with a poor prognosis in various diseases, such as cardiovascular diseases, solid cancers, and infections.

Increased NLR during COVID-19 could be a result of the expression of inflammatory cytokines, an unusual increase in pathological low-density neutrophils, and the upregulation of genes involved in the lymphocyte cell death pathway caused by SARS-CoV-2 infection. ^36^ Furthermore, two studies ^37^^,^^38^ have proposed integrating the NLR into nomograms to prove the prognostic value of this biomarker.

Our study showed that for a threshold ≥ 5, the sensitivity and specificity of NLR in predicting critical status on Day 7 were 68.7% and 51%, respectively. This sensitivity was 56.52% in the study by Liu and al. ^38^ Therefore, NLR is an objective, simple, and inexpensive parameter that could be used in routine practice as a prognostic biomarker.

Thrombotic complications and coagulopathy represent a major event during SARS-CoV-2 infection. An increase in D-dimer and fibrinogen levels and a decrease in prothrombin time indicate a state of hypercoagulability. Many studies have shown that high levels of D-dimer are associated with severe ^39^^,^^40^ and critical ^10^ forms, the need for intensive care management, ^2^^,^^18^ and in-hospital mortality due to COVID-19 pneumonia. ^28^^,^^41^ This hypercoagulability in COVID-19 patients could result from several mechanisms ^42^: in viral infections, there is often an unbalance between pro-inflammatory response and anti-inflammatory response; this can lead to endothelial cell dysfunction and excessive thrombin production; hypoxia contributes to thrombosis by increasing blood viscosity.

Increased D-dimer levels indicate that the fibrinolytic system is activated in COVID-19 patients. Furthermore, the increased release of cytokines during viral infections stimulates coagulation cascade. ^43^ A reduction in D-dimer levels was observed in recovered patients, independently of anticoagulant treatment, while a continuous increase in D-dimer levels was predictive of a higher risk of thrombosis and unfavorable outcomes. ^42^^,^^44^ Thus, D-dimers are an early and reliable marker for predicting a poor prognosis in COVID-19 hospitalized patients. ^42^

In our study, thromboembolic events occurred among 11 patients. The incidence of pulmonary embolism (PE) in COVID-19 patients is still unknown and likely underestimated. ^45^ Bilaloglu and al. reported a prevalence of PE of 3.2% in a study involving 3,334 COVID-19 patients in New York, ^46^ while according to a French study, ^47^ the incidence of PE was 23.7%. This difference could be explained by differences in disease severity, patient characteristics, and the limited use of CT angiography among patients.

### Biochemical biomarkers

Biochemical tests findings, showed that the critical group of patients had elevated level of CRP, D-Dimer, creatinine, lactate dehydrogenase, creatine kinase, troponin and NT-Pro-BNP, which corroborates the conclusion made by Feng and al. ^40^

The CRP was elevated (>8 mg/L) in 93.5% of patients with an average of 110 ± 73 mg/L. We noted that the CRP on admission was significantly higher in the critical group (p=0.047). In multivariate analysis, an elevated CRP level was correlated with an increased risk of progressing to a critical condition on the 7th day of admission. Many studies have shown that elevated CRP levels are correlated with critical forms, ^10^^,^^39^ disease progression, ^48^ and mortality from COVID-19 pneumonia. ^21^ As known, CRP is a non-specific marker of inflammation induced by interleukin-6 (Il-6) secretion. In clinical practice, it is used as a biomarker for various inflammatory and infectious conditions. High CRP levels have been directly correlated with the inflammation’s degree and disease severity. ^49^ Moreover, CRP levels among dead COVID-19 patients were decuple higher than survivors. ^50^

A meta-analysis of 20 studies including 4,843 COVID-19 patients and focusing on the clinical utility of CRP, ^51^ emphasized that high CRP level was associated with a fourfold higher risk of an unfavorable outcome (p<10 ^−3^). In fact, at the early stages of COVID-19 infection, an increase in CRP was directly associated with the development of lung lesions, reflecting the severity of the disease. ^51^^,^^52^ Furthermore, Ali and al. ^53^ demonstrated that CRP level could predict disease worsening among non-severe cases, indicating 5% risk of progressing to a severe form for each unit increase in the CRP rate. Added to that, the CRP level has also been reported as a reliable biomarker for treatment responses in COVID-19 patients ^19^; in fact, this marker could be used to select patients who would benefit from treatment with tocilizumab, another IL-6 receptor inhibitor similar to sarilumab. ^54^^,^^55^

Our findings indicated that NLR and CRP are good predictors of unfavorable outcome. The reported excellent accuracy of these parameters in the prediction of COVID-19 patients’ outcome corroborates findings of the studies of Yang and al. ^31^ and. Liu and al. ^38^

We found that blood creatinine (p=0.026) and urea levels (p=0.061) were higher in the critical group. Furthermore, urea levels were significantly higher in elderly patients (> 70 years old) (p<10 ^−3^) and in patients with high blood pressure (p=0.012). Elevated blood urea (≥ 7 mmol/L) was noted in 46.6%. These findings align with the literature, where it has been demonstrated that elevated blood creatinine and urea values are associated with severe disease, unfavorable prognosis, and significant mortality. ^41^^,^^56^^,^^57^ A Tunisian study conducted between September and December 2020, ^58^ illustrated that acute renal failure was associated with poor outcome (OR:6.7) for patients hospitalized in ICU. The mechanism of renal involvement in COVID-19 is likely to be multifactorial. ^56^ It involves direct cytopathic effects on kidney tissue by the virus leading to renal cell necrosis as well as indirect damage by cytokines and metabolites induced by hypoxia, shock, or rhabdomyolysis.

Increased liver enzymes level was found in 39.5%, especially in male patients (p<10 ^−3^), while AST and ALT rates did not vary between both groups. Numerous studies have demonstrated the association between high transaminase levels and the severity of the disease, ^39^^,^^40^^,^^57^^,^^59^ transfer to the ICU ^2^^,^^18^^,^^60^^,^^61^ and death ^28^^,^^41^ due to COVID-19. Jamoussi and al. ^62^ have concluded that IL-6 ≥ 20 pg/ml, CK < 107 UI/L,AST < 30UI/L were independent risk factors for mortality among patients admitted in ICU between September and December 2020 in Tunisia. Moreover, the good accuracy of AST and ALT as a predictor of ICU admission have been clearly shown with AUC>0.7. ^63^ Added to that, Malik and al. demonstrated in their meta-analysis ^51^ that high levels of AST and ALT (> 40 IU/L) were associated with a threefold higher risk of a poor prognosis. Some studies have shown that COVID-19 only transiently increases transaminases. Cytolysis is rather due to liver damage secondary to systemic inflammatory processes, hypoxia, or to the use of hepatotoxic drugs, especially antivirals such as Lopinavir and Ritonavir during patient management. ^51^^,^^64^ However, viral RNA has been detected in the liver at high titers, exceeding viremia, during autopsies, suggesting that SARS-CoV-2 hepatic infection can contribute to elevation of transaminase levels in patients with severe forms of COVID-19. ^44^^,^^65^

Regarding rhabdomyolysis markers, we found an elevation of LDH and CK levels among 98% and 24% of our patients, respectively. We also demonstrated that muscle lysis enzymes were significantly higher in critical group patients. Our data were consistent with the literature, where it has been shown that high levels of LDH and CK were associated with the severity ^5^^,^^66^ and progression ^67^ of the disease, transfer to ICU, and mortality. ^57^ Jamoussi A ^62^ from COVID-19. In a meta-analysis assessing the prognostic value of LDH levels, ^41^ it was found that elevated LDH levels were associated with a risk of mortality (OR: 16) and severe disease (OR: 6). Furthermore, a study conducted on COVID-19 patients ^68^ showed that increased LDH levels at the early stage of the disease can predict lung damage and severe cases of COVID-19. These high LDH levels may result from decreased tissue oxygenation leading to stimulation of the glycolytic pathway or from damage of multiple organs in case of multi-organ failure. ^51^ Additionally, severe infections can induce cytokine-mediated tissue damage and LDH release. ^69^

Concerning cardiac injury markers, we found that elevated levels of cardiac troponins were correlated with critical conditions (p=0.001). Indeed, troponins have a prognostic value in sepsis ^70^ and have been proposed as severity markers ^18^ of the disease and predictors of COVID-19 mortality. ^28^^,^^41^ These disturbances in cardiac enzymes can result from viral myocarditis, myocardial damage caused by cytokines or microangiopathies and coronary spasms secondary to hypoxia. ^71^

Furthermore, according to our data, we observed an increase in NT-Pro-BNP levels among 25 patients (5.5%), indicating a left heart failure. Additionally, the mean NT-Pro-BNP value was higher in the critical group of patients (p=0.011). The mechanisms of heart failure can be attributed to an imbalance between increased cardiac output and reduced oxygen supply, with the possibility for type 2 myocardial infarction. ^72^ According to Li and al., ^39^ NT-Pro-BNP>500pg/L were significantly associated with severe forms of COVID-19, and Hong and al., ^10^ demonstrated that critically ill patients had higher NT-Pro-BNP levels (p=0.002). Therefore, cardiac biomarkers, including troponins and NT-Pro-BNP, can reflect cardiovascular involvement in COVID-19, since they are independent risk factors for poor prognosis and mortality. ^72^^,^^73^

### Strength and limitations

Our study has shown that some biochemical and CBC tests are important in predicting COVID-19 patients’ need for ICU care. Specifically, laboratory tests that should be prioritized to determine patient risk of developing severe COVID-19 pneumonia, include lymphopenia, NLR, thrombocytopenia, CRP, D-Dimer, creatinine, LDH, CK, troponin and NT-Pro-BNP. NLR is most preferred as it was noted to be a very good test.

However, limitations should be considered in the interpretation of our findings. First, our study is retrospective and monocentric. Second, some biological parameters were missing in the medical records and other specialized tests are not commonly performed in our hospital such as Il-6 and Procalcitonin given their cost. Also, the lack of systematic data on blood gas analysis on Day 7, did not allow us to evaluate PaO _2_/FiO _2_ ratio, to define ARDS. Besides, the study did not consider the influence of pre-existing health conditions.

Despite its limitations, this study has provided insights into laboratory parameters that can be used to predict the severity of COVID-19 cases allowing prediction of severe illness at the time of admission. It is therefore recommended that healthcare providers consider these parameters in making evidence-based decisions regarding patient management especially where there are limited ICU facilities.

It is worth mentioning that most of reported studies were conducted in different geographical and temporal contexts, some studies were conducted during the same period as our study. ^58^^,^^62^ Indeed, COVID-19 pandemic waves showed significant time lags across the world due to several factors, including health control measures, vaccination rates, population density, and circulating viral strains. For example, while some countries experienced a severe first wave at the beginning of 2020, other regions saw a peak in cases later in the year or even in 2021 with the emergence of more transmissible variants like Delta. ^74^ In Tunisia, the first wave ranged from March to June 2020 and was characterized by small numbers of circulating lineages. The second wave ranged from July 2020 to January 2021 and was characterized by a higher genetic diversity with the circulation of at least 20 different lineages. The third wave ranged from February to May 2021 and was characterized by the emergence of variants of concern (VOCs) and variants of interest (VOI), such as Alpha who rapidly became the predominant variant. The fourth wave started on June/July 2021, the overwhelming majority of detected lineages was Delta. ^1^ Consequently, our study included several variants of the virus, which impacts the clinical presentation and the outcome of the infection. However, we believe that although the period of our study is lagged compared to the studies cited in the discussion, our results remain relevant for assessing the prognosis of patients with COVID-19, regardless of the variant.

## Conclusion

In conclusion, we have identified certain biological markers that can be used to assess the risk of COVID-19 pneumonia progressing to a critical state.

Particularly; lymphopenia, high NLR, thrombocytopenia, and elevated CRP level are all significantly associated with poor prognosis.

Therefore, in patients hospitalized with moderate to severe forms, we recommend close monitoring of these biomarkers, since immune suppression, heightens inflammation and alters host's adaptive response capabilities.

Since the beginning of the pandemic, it has been scientifically important to analyze the discriminatory capacity of hematological, biochemical, inflammatory and immunological biomarkers in patients with COVID-19, with or without a severe or critical form. Determining risk categories after the diagnosis of COVID-19 is essential for better resource allocation, improved clinical management and prevention of serious complications.

### Ethical approval and consent

This retrospective study received approval from the Ethics Committee of University Hospital Center Mongi Slim, under approval number 57/2023 on Friday, 22 December 2023.

We followed strict ethical committee guidelines that allowed for exemption of consent (institute policy), due to the non-intrusive nature of the study and the use of non-identified data to ensure confidentiality and anonymity of participants. All personally identifiable data were anonymized prior to analysis to protect individuals’ privacy. We also carefully assessed the risks and benefits of our research, ensuring to minimize the former and maximize the latter for participants and the scientific community Policies regarding the future use of data and their potential sharing with other researchers were strictly established in accordance with ethical guidelines.

## Data Availability

The data supporting the findings of this study are not publicly available due to ethical concerns. However, fully de-identified data will be made available upon request from reviewers and readers. Interested parties should contact the corresponding author at donia.belkhir@fmt.utm.tn to request access to the data. Data requests will be reviewed in accordance with the local ethical committee.
