# Supplementary material for: Laboratory findings predictive of critical illness in hospitalized COVID-19 patients in Tunisia
Source: F1000Res. 2024 Nov 18;13:918. Originally published 2024 Aug 12. [Version 2] doi: 10.12688/f1000research.151333.2 (PMC11628936; doi:10.12688/f1000research.151333.2)
Supplement: Peer review history

# Reviewer report: Reviewer response for version 2

**DOI:** 10.5256/f1000research.174176.r344478

**Published:** 2024-12-09

**Authors:** Santiago J Ballaz

**Affiliations:** 1 Universidad Espíritu Santo, Samborondón, Ecuador

To the attention of Belkhir and colleagues,

 My concern with the manuscript is the low sensitivity (CRP, NLR, and platelets) of the laboratory findings. These variables have relatively low AUC/ROC values, making it difficult to make inferences regarding their predictive efficacy. The parameters obtained from the Youden test are clinically irrelevant, even though the ORs and AUCs are statistically significant.

 Nevertheless, the research work is rescuable. There are a series of indices combined laboratory test ratios like the SII index and the red blood cell distribution width-to lymphocyte ratio (RLR) that should also be included in the research. Expanding your manuscript with laboratory results pertaining to the subject of study shouldn't be a huge difficulty.

 Minor points: Please mention the Youden test in the statistics section. Also define how you classify “moderate COVID-19 pneumonia.”

Is the work clearly and accurately presented and does it cite the current literature?

Yes

If applicable, is the statistical analysis and its interpretation appropriate?

Partly

Are all the source data underlying the results available to ensure full reproducibility?

Yes

Is the study design appropriate and is the work technically sound?

Partly

Are the conclusions drawn adequately supported by the results?

Partly

Are sufficient details of methods and analysis provided to allow replication by others?

Yes

Reviewer Expertise:

COVID-19 research

I confirm that I have read this submission and believe that I have an appropriate level of expertise to confirm that it is of an acceptable scientific standard, however I have significant reservations, as outlined above.

## References

[1] : Sex-Dependent Performance of the Neutrophil-to-Lymphocyte, Monocyte-to-Lymphocyte, Platelet-to-Lymphocyte and Mean Platelet Volume-to-Platelet Ratios in Discriminating COVID-19 Severity. *Front Cardiovasc Med* .2022;9: 10.3389/fcvm.2022.822556 822556 10.3389/fcvm.2022.822556 35463770 PMC9023889

[2] : Evaluation of the red blood cell distribution width-to-lymphocyte ratio (RLR) and red blood cell distribution width-to-platelet ratio (RPR) as predictors of COVID-19 in-hospital mortality. *Anaesthesia Critical Care & Pain Medicine* .2023;42(5) : 10.1016/j.accpm.2023.101274 10.1016/j.accpm.2023.101274 37473459

[3] : Predictive Value of the Platelet Times Neutrophil-to-Lymphocyte Ratio (SII Index) for COVID-19 In-Hospital Mortality. *EJIFCC* .2023;34(2) :167-173 37455846 PMC10349308

# Reviewer report: Reviewer response for version 2

**DOI:** 10.5256/f1000research.174176.r341412

**Published:** 2024-11-19

**Authors:** Stelvio Tonello

**Affiliations:** 1 University of Piemonte Orientale (UPO), Novara, Italy

I have checked the new version of this manuscript and I think that is good and suitable for indexing.

Is the work clearly and accurately presented and does it cite the current literature?

Yes

If applicable, is the statistical analysis and its interpretation appropriate?

Yes

Are all the source data underlying the results available to ensure full reproducibility?

No source data required

Is the study design appropriate and is the work technically sound?

Partly

Are the conclusions drawn adequately supported by the results?

Yes

Are sufficient details of methods and analysis provided to allow replication by others?

Partly

Reviewer Expertise:

NA

I confirm that I have read this submission and believe that I have an appropriate level of expertise to confirm that it is of an acceptable scientific standard.

# Reviewer report: Reviewer response for version 1

**DOI:** 10.5256/f1000research.165971.r314706

**Published:** 2024-11-12

**Authors:** Vignesh Mariappan

**Affiliations:** 1 Laboratory of virology,Disease Modelling and Transmission section, Rocky Mountain Laboratories, Hamilton, MT, 59840, MT, USA; 2 MGMARI, Sri Balaji Vidyapeeth University, Puducherry, Puducherry, India

In this manuscript, Belkhir and the team aimed to identify potential prognostic biomarkers for predicting severe disease outcomes in COVID-19 patients. The study compared two groups -critical and non-critical patients who were infected during the first wave of SARS-CoV-2 virus. Their data suggest differences in the levels of certain clinical biological indicators and their association with the critical group. Overall, the study is well-designed and employs robust statistical analysis.

**Overall Comments**

 Since 2020, numerous research studies and reports have been indexed regarding potential biomarkers for the early prediction of COVID-19 disease outcomes.

1. Given the emergence of several variants of SARS-CoV-2, do the authors believe their findings could be applicable for evaluating the prognosis of COVID-19 patients infected with different variants?

Comments:

**Introduction**

1. The introduction does not clarify the relationship between study markers and SARS-CoV-2 infection.

**Methodology**

1. Why was the 7 ^th^ day of hospitalization chosen as an outcome measure?
2. Are patients transferred to the intensive care unit before or on Day 7 classified as critical or non-critical? This classification is repeated for both outcome measure, which is bit confusing.
3. If a patient from the non-critical group progresses to a critical state is that patients included in the critical group.
4. The definition of ARDS could be removed for brevity.
5. Please condense the study section into 2-3 paragraphs.
6. More details about D-dimer, CRP, Troponin and NT-Pro-BNP measurement should be further explained has a separate section each?

**Results**

1. What clinical symptom is used to calculate the time of onset of symptoms to admission in the COVID-19 unit?
2. In Table 1, the authors indica that 11 patients from the non-critical group were transferred to the ICU. Based on the study’s critical group outcome measure, were these 11 patients included in the critical group?
3. In Table 3, please remove the word “elevated” from the side headings.
4. Does “N” in the side heading of Table 3 refers to mean or count?

**Discussion and limitation**

1. The discussion and limitation section is lengthy and may lose the reader’s interest. Consider streamlining this section to enhance engagement.

**Conclusion**

1. The authors provided a generalized statement in the conclusion/ Please specify the major findings concisely. Please modify the same in abstract section.

Is the work clearly and accurately presented and does it cite the current literature?

Yes

If applicable, is the statistical analysis and its interpretation appropriate?

Yes

Are all the source data underlying the results available to ensure full reproducibility?

No source data required

Is the study design appropriate and is the work technically sound?

Partly

Are the conclusions drawn adequately supported by the results?

Yes

Are sufficient details of methods and analysis provided to allow replication by others?

Partly

Reviewer Expertise:

Biomarker Identification, Virology, and Vaccines

I confirm that I have read this submission and believe that I have an appropriate level of expertise to confirm that it is of an acceptable scientific standard, however I have significant reservations, as outlined above.

# Response

**Published:** 2024-11-13

**Authors:** Donia Belkhir

**Affiliations:** Pulmonology, University Hospital Center Mongi Slim, La Marsa, Tunis, Tunisia

We would like to thank you for reviewing our article, and for your valuable comments, which have contributed to improving the quality of our manuscript. We have addressed each of your suggestions and comments in detail, as outlined below:

**Overall comments:**

 1- Given the emergence of several variants of SARS-CoV-2, do the authors believe their

 findings could be applicable for evaluating the prognosis of COVID-19 patients infected with different variants?

 Response: Yes, We have added in the limitations section that we took into consideration the fact that our study included several variants of the virus, which impacts the clinical presentation and the outcome of the infection. However, we believe that although the period of our study is lagged compared to the studies cited in the discussion, our results remain relevant for assessing the prognosis of patients with COVID-19, regardless of the variant.

**Introduction:**

 1) The introduction does not clarify the relationship between study markers and SARS-CoV-2 infection: 

 Response:We added a paragraph in the introduction section, clarifying the relationship between SARS-COV2 infection and the study of biomarkers.

**Methodology:**

 * Why was the 7th day of hospitalization chosen as an outcome measure?

 Response: Several studies have shown the variation over time of the various markers, accompanying the “cytokine storm” that precedes the onset of ARDS, which usually occurs after 8 to 12 days from the onset of symptoms. Hence, we chose the 7th day after hospitalization, approximately corresponding to the 14th day after symptom onset, as the threshold for classifying patients into critical or non-critical groups.

 We added a paragraph explaining the phenomenon of cytokine storm and our choice of the 7 ^th^ day of hospitalization.

 *Are patients transferred to the intensive care unit before or on Day 7 classified as critical or non-critical? This classification is repeated for both outcome measure, which is bit confusing.

 Response: Patients transferred to the intensive care unit before or on day 7 were classified as critical. (It was incorrect to mention this phrase in the "non-critical" paragraph, we corrected it).

 *If a patient from the non-critical group progresses to a critical state is that patients included in the critical group:

 Response: Patients who developed critical state after day 7 were not considered among the critical group.

 * The definition of ARDS could be removed for brevity 

  Response: This section was removed.

 *Please condense the study section into 2-3 paragraphs

 Response: This section was condensed in 3 paragraphs.

 *More details about D-dimer, CRP, Troponin and NT-Pro-BNP measurement should be

 further explained as a separate section each

 Response:  We added a short description to detail each marker

**Results:**

 * What clinical symptom is used to calculate the time of onset of symptoms to admission in the COVID-19 unit? 

 Response: Any of the 6 symptoms listed in Table 1 

 * In Table 1, the authors indicate that 11 patients from the non-critical group were transferred to the ICU. Based on the study’s critical group outcome measure, were these 11 patients included in the critical group? 

 Response: Patients transferred to ICU after day 7 were not considered as critical.

 * In Table 3, please remove the word “elevated” from the side headings. 

 Response: The word “Elevated” was replaced by “Increased” (Please note that the items in this table correspond to marker abnormalities, and the number of patients with abnormalities, and not to their levels).

 * Does “N” in the side heading of Table 3 refers to mean or count?

 Response: N refers to the count.

**Discussion and limitation**

  1. The discussion and limitation section is lengthy and may lose the reader’s interest. Consider streamlining this section to enhance engagement.

 Response: We have removed some parts that do not affect the scope of the discussion.

**Conclusion**

 1. The authors provided a generalized statement in the conclusion/ Please specify the major findings concisely. Please modify the same in abstract section

 Response:Some specifications related to the majors findings were added in this section as well as in the abstract

# Reviewer report: Reviewer response for version 1

**DOI:** 10.5256/f1000research.165971.r319441

**Published:** 2024-09-04

**Authors:** Stelvio Tonello

**Affiliations:** 1 University of Piemonte Orientale (UPO), Novara, Italy

The paper presented by Belkir and colleagues offers a glimpse into the possibility of identifying new parameters and new biomarkers for COVID-19 patients with severe syndrome.

 The study was carried out by analyzing patients who had contracted the infection during the first wave of covid-19

 the work presents excellent insights for analysis.

 1) the inclusion and exclusion parameters are defined but it is not clear whether the patients analyzed were at their first infection or not

 2) when the data are analyzed and discussed, no reference is made to the studies carried out in previous years

 3 The insufficiency of some data, although justified, makes the paper lacking in information that could be very interesting

 In conclusion, despite having some limitations and missing a paragraph on the status of the studies carried out over the same period (this paragraph is required), publication is recommended

Is the work clearly and accurately presented and does it cite the current literature?

Yes

If applicable, is the statistical analysis and its interpretation appropriate?

Yes

Are all the source data underlying the results available to ensure full reproducibility?

No source data required

Is the study design appropriate and is the work technically sound?

Partly

Are the conclusions drawn adequately supported by the results?

Yes

Are sufficient details of methods and analysis provided to allow replication by others?

Partly

Reviewer Expertise:

Pharmacology internal medicin Virology

I confirm that I have read this submission and believe that I have an appropriate level of expertise to confirm that it is of an acceptable scientific standard, however I have significant reservations, as outlined above.

# Response

**Published:** 2024-11-05

**Authors:** Donia Belkhir

**Affiliations:** Pulmonology, University Hospital Center Mongi Slim, La Marsa, Tunis, Tunisia

We would like to thank you for reviewing our article, and for your valuable comments, which have contributed to improving the quality of our manuscript. We have addressed each of your suggestions and comments in detail, as outlined below:

 1) The inclusion and exclusion parameters are defined but it is not clear whether the patients analyzed were at their first infection or not: 

 -All patients were at their first infection. We added this information in the methods section

 2) When the data are analyzed and discussed, no reference is made to the studies carried out in previous years:

 -Some of our results are consistent with studies of MERS and SARS, and we have reported certain findings in these two diseases.

 *In conclusion, despite having some limitations and missing a paragraph on the status of the studies carried out over the same period (this paragraph is required): 

 -We appreciate your suggestion. As per your recommendation, we have added the results of two Tunisian studies conducted during the same period as ours. We have also added a paragraph explaining the variability between studies in terms of periods, waves and variants.

 We believe that this does not affect the relevance of our results, regardless of the period and the variant.  

 .
